# Risikofaktoren für postoperative Hypoxämie während des Transportes in den Aufwachraum und Einfluss von Transport-Monitoring: Eine retrospektive Propensity-Score-gematchte Datenbankanalyse

**DOI:** 10.1007/s00101-023-01296-y

**Published:** 2023-06-09

**Authors:** Katharina Haller, Ralf Felix Trauzeddel, Sascha Treskatsch, Christian Berger

**Affiliations:** Klinik für Anästhesiologie mit Schwerpunkt operative Intensivmedizin, Charité – Universitätsmedizin Berlin, corporate Member of Freie Universität and Humboldt Universität zu Berlin, Hindenburgdamm 30, 12203 Berlin, Deutschland

**Keywords:** Postoperative Komplikationen, Perioperatives Risikomanagement, Sauerstoff, Patientensicherheit, Pulsoxymetrie, Postoperative complications, Perioperative risk management, Oxygen, Patient safety, Pulse oximetry

## Abstract

**Hintergrund:**

Auf Transportwegen innerhalb eines Zentral-OP nach der Narkoseausleitung in den Aufwachraum (AWR) sind Patienten hypoxämiegefährdet. Spezifische Risikofaktoren sind jedoch nicht abschließend geklärt, und einheitliche Empfehlungen zur Überwachung der Vitalparameter bei Transporten innerhalb eines OP-Komplexes existieren nicht. Ziel dieser retrospektiven Datenbankanalyse war es, Risikofaktoren für eine Hypoxämie auf diesen Transporten zu identifizieren und zu prüfen, ob die Verwendung eines Transport-Monitorings (TM) den initialen Wert der peripher-venösen Sauerstoffsättigung (S_p_O_2_) im AWR beeinflusst.

**Material und Methoden:**

An einem retrospektiv extrahierten Datensatz von Eingriffen in Allgemeinanästhesie innerhalb eines Zentral-OP einer Universitätsklinik von 2015 bis 2020 wurden Risikofaktoren für eine initiale Hypoxämie im AWR (S_p_O_2_ < 90 %) mittels multivariater Analyse ermittelt. Nach Aufteilung des Datensatzes in Patienten ohne TM (Gruppe OM) und mit TM (Gruppe MM) und Propensity Score Matching wurde der Einfluss des TM untersucht.

**Ergebnisse und Diskussion:**

Acht Risikofaktoren für eine initiale Hypoxämie im AWR konnten identifiziert werden: Alter > 65 Jahre, body mass index (BMI) > 30 kg/m^2^, chronisch obstruktive Lungenerkrankung (COPD), intraoperativer Beatmungsdruck-Hub (∆p) > 15 mbar und positiver endexpiratorischer Druck (PEEP) > 5 mbar, intraoperative Gabe eines lang wirksamen Opioids, erste präoperative S_p_O_2_ < 97 % sowie nach Anästhesieausleitung letzte im OP gemessene S_p_O_2_ < 97 %. Bei 90 % aller Patienten lag mindestens ein Risikofaktor für eine postoperative Hypoxämie vor. Bei Vorliegen von Risikofaktoren geht die Verwendung eines TM mit einer geringeren initialen Desaturierung (MM: 97 [94; 99] %, OM: 96 [94; 98] %, *p* < 0,001) im AWR einher. Demnach erscheint eine konsequente Nutzung von TM auch auf kurzen Transporten innerhalb eines zusammenhängenden OP-Komplexes sinnvoll.

**Zusatzmaterial online:**

Die Online-Version dieses Beitrags (10.1007/s00101-023-01296-y) enthält eine zusätzliche Tabelle mit den Daten zu Abb. 2.

## Abstract (en)

**Background:**

Within a central operating room area, after general anesthesia (GA) patients are at risk of hypoxemia during transport to the postanesthesia care unit (PACU); however, specific risk factors have not been conclusively clarified and uniform recommendations for monitoring vital signs during transport within a central operating room area complex do not exist. The purpose of this retrospective database analysis was to identify risk factors for hypoxemia during this transport and to determine whether the use of transport monitoring (TM) affects the initial value of peripheral venous oxygen saturation (S_p_O_2_) in the PACU.

**Material and methods:**

This analysis was performed on a retrospectively extracted dataset of procedures in GA within a central operating room area of a tertiary care hospital from 2015 to 2020. The emergence from GA was conducted in the operating room with subsequent transport to the PACU. The transport distance was between 31 and 72 m. Risk factors for initial hypoxemia in the PACU, defined as peripheral oxygen saturation (S_p_O_2_) below 90%, were determined using multivariate analysis. After splitting the dataset into patients without TM (group OM) and with TM (group MM) and propensity score matching, the influence of TM on initial S_p_O_2_ and the Aldrete score after arrival in the PACU were examined.

**Results and discussion:**

From a total of 22,638 complete datasets included in the analysis, 8 risk factors for initial hypoxemia in PACU were identified: age > 65 years, body mass index (BMI) > 30 kg/m^2^, chronic obstructive pulmonary disease (COPD), intraoperative airway driving pressure (∆p) > 15 mbar and positive endexpiratory pressure (PEEP) > 5 mbar, intraoperative administration of a long-acting opioids, first preoperative S_p_O_2_ < 97%, and last S_p_O_2_ < 97% measured after emergence from anesthesia before transport. At least 1 risk factor for postoperative hypoxemia was present in 90% of all patients. After propensity score matching, 3362 datasets per group remained for analysis of the influence of TM. Patients transported with TM revealed a higher S_p_O_2_ at PACU arrival (MM 97% [94; 99%], OM 96% [94; 99%], *p* < 0.001). In a subgroup analysis, this difference between groups remained in the presence of one or more risk factors (MM 97% [94; 99%], OM 96% [94; 98%], *p* < 0.001, *n* = 6044) but was not detectable in the absence of risk factors for hypoxemia (MM 97% [97; 100%], OM 99% [97; 100%], *p* < 0.393, *n* = 680). Furthermore, the goal of an Aldrete score > 8 at PACU arrival was achieved significantly more often in monitored patients (MM 2830 [83%], OM: 2665 [81%], *p* = 0.004). Critical hypoxemia (S_p_O_2_ < 90%) at PACU arrival had an overall low occurrence within propensity matched datasets and showed no difference between groups (MM: 161 [5%], OM 150 [5%], *p* = 0.755). According to these results, consistent use of TM leads to a higher S_p_O_2_ and Aldrete score at PACU arrival, even after a short transport distance within an operating room area. Consequently, it appears to be reasonable to avoid unmonitored transport after general anesthesia, even for short distances.

## Hinführung zum Thema

Der postoperative Patiententransport vom OP in den Aufwachraum ist ein möglicher kritischer Moment für eine Hypoxämie. Daher stellen sich die Fragen nach spezifischen Risikofaktoren für eine Hypoxämie in diesem Zeitraum, und ob ein Transport-Monitoring (TM) einen Einfluss auf die Häufigkeit des Auftretens hat.

## Hintergrund und Fragestellung

Patienten sind perioperativ und insbesondere während und nach der Ausleitungsphase einer Allgemeinanästhesie mit dem daraufhin folgenden Transport in den Aufwachraum (AWR) der Gefahr einer Hypoxämie ausgesetzt. Hierbei kann es in bis zu 20 % der Fälle zum Abfall der peripher-venösen Sauerstoffsättigung (S_p_O_2_) unter 90 % kommen [1, 4, 14, 26]. Dabei stellt die postoperative Hypoxämie eine gefährliche Komplikation dar, die zu schwerwiegenden Ereignissen wie Arrhythmien oder myokardialen Ischämien führen kann [11].

Frühere Arbeiten konnten niedrige präoperative S_p_O_2_, höheren Body-Mass-Index (BMI), Alter, ASA-Status, „driving pressure“ der Beatmung, Wahl des Opioids und des Relaxans als unabhängige Einflussgrößen für das Auftreten einer Hypoxämie identifizieren [4, 13, 14]. Insgesamt sind diese Erkenntnisse nicht einheitlich und auch im Hinblick auf sich ändernde Anästhesieverfahren nicht abschließend bewertbar. Zudem gibt es Hinweise, dass eine reine Patientenbeobachtung auf solch kurzen Transporten nicht ausreichend ist [4]. Klare, einheitliche Handlungsanweisungen zum Monitoring auf kurzen Transportwegen sind aktuell nicht vorhanden, und die Möglichkeit der kurzfristigen Unterbrechung eines kontinuierlichen Monitorings wird für kurze Transportwege eingeräumt [3].

Ziel dieser retrospektiven, monozentrischen Datenbankanalyse war es daher, aktuelle Risikofaktoren für das Auftreten einer Hypoxämie nach Anästhesieausleitung zu identifizieren sowie zu evaluieren, ob die Anwendung eines TM zu einer Beeinflussung der initialen S_p_O_2_ bei Ankunft im AWR führt.

## Material und Methoden

Diese retrospektive, monozentrische, Propensity-Score-gematchte Datenbankanalyse wurde am Campus Benjamin Franklin (CBF), Charité – Universitätsmedizin Berlin, nach positivem Ethikvotum der Ethikkommission der Charité – Universitätsmedizin Berlin (EA-Votum: EA4/239/19) sowie positivem Datenschutzvotum durchgeführt. Alle im Zentral-OP (ZOP) durchgeführten Eingriffe in Allgemeinanästhesie im Zeitraum vom 01.07.2015 bis zum 01.07.2020 wurden für die Auswertung gescreent. Der ZOP beinhaltet 15 OP mit Wegstrecken zwischen minimal 31 und maximal 72 m in den AWR. Das Eingriffsspektrum beinhaltet Eingriffe in sämtlichen operativen Fachdisziplinen mit Ausnahme der Kardiochirurgie und Geburtshilfe. Die Narkoseführung ist in Standard Operating Procedures (SOP) festgehalten, in denen zum Zeitpunkt der Auswertung die Anästhetika Desfluran, Sevofluran und Propofol sowie die Opioide Sufentanil, Remifentanil und Fentanyl zur Anwendung kamen. Als Muskelrelaxanzien bei Intubationsnarkose wurden Cisatracurium, Mivacurium oder Rocuronium verwendet.

Alle vollständigen Patientendatensätze (≥ 18 Jahre) im Untersuchungszeitraum wurden aus dem elektronischen Dokumentationssystem (Fa. COPRA System, Sasbachwalden, Deutschland) eingeschlossen und ausgewertet. Unvollständige Datensätze sowie Datensätze ohne Allgemeinanästhesie oder ohne Aufenthalt im AWR wurden ausgeschlossen. Der Gesamtdatensatz (ungemachte Gesamtkohorte) wurde zur Identifizierung von Risikofaktoren für Hypoxämie nach Transport aus dem OP mittels binärlogistischer Regressionsanalyse auf das Auftreten einer Hypoxämie (S_p_O_2_ < 90 %) zum Zeitpunkt der Ankunft im AWR untersucht. Zur Abschätzung der Stärke des Einflusses wurden die Odds Ratios mit den dazu gehörigen 95 %-Konfidenzintervallen berechnet.

In einem zweiten Schritt wurde der Gesamtdatensatz in eine „Kontrollgruppe ohne TM“ (OM; Zeitraum 01.07.2015 bis 30.06.2017) und eine „Interventionsgruppe mit TM“ (MM; Zeitraum 01.07.2018 bis 31.06.2020) aufgeteilt. Diese Aufteilung wurde aufgrund einer Verfahrensänderung am CBF mit Einführung eines TM, bestehend aus Blutdruckmessung, S_p_O_2_ und Elektrokardiogramm, Ende 2017 für Transporte aus dem OP in den AWR gewählt. Vor diesem Zeitpunkt bestand kein TM auf diesen Wegen. Die Datensätze zwischen 01.07.2017 bis 30.06.2018 wurden als „Wash-out“-Periode zur Verfahrenstrennung nicht eingeschlossen.

Zwischen OM- und MM-Datensatz wurde ein 1:1-Propensity-Score-Matching (PSM) nach Alter, Geschlecht und BMI (kategorial als < 25 kg/m^2^, 25–30 kg/m^2^ und > 30 kg/m^2^) sowie den übergreifenden kategorialen anästhesierelevanten Scores ASA-Status [20], N‑Klassifikation [6] und Operationsrisiko [10] durchgeführt und hinsichtlich der initialen S_p_O_2_ im AWR sowie der Differenz zur präoperativen Messung (∆S_p_O_2_) verglichen. Zusätzlich wurden Häufigkeit des Auftretens von Hypoxämie (definiert als mild: 90–86 %, moderat: 85–81 %, schwer: < 81 %), Atemfrequenz und Aldrete-Score [2], Häufigkeit der Aufnahme aus dem AWR auf die Intensivstation, Krankenhausverweildauer sowie die Dauer von Ausleitung mit Patientenumlagerung in das Patientenbett mit folgendem Transport in den AWR (Gesamttransportprozess) anhand des Ende des Monitorings im OP und erster S_p_O_2_-Messung im AWR ausgewertet.

### Statistik

Die statistische Auswertung erfolgte mit R Version 1.3.1093 [21]. Normalverteilung wurde mittels Quantil-Quantil-Diagrammen und Histogrammen überprüft. Nicht normal verteilte Daten werden als Median und Interquartilbereich, normal verteilte als Mittelwert ± Standardabweichung und qualitative Merkmale als Anzahl (Prozent) dargestellt.

Bei der binär logistischen Regressionsanalyse für das Auftreten einer Hypoxämie wurden folgende Faktoren berücksichtigt: Alter > 65 Jahre, Geschlecht, BMI > 30 kg/m^2^, ASA > II, chronisch obstruktive Bronchialerkrankung (COPD) Stadien I–IV nach GOLD, obstruktives Schlafapnoe-Syndrom (OSAS), Herzinsuffizienz, Belastbarkeit < 4 metabolische Äquivalente (MET), Notfalleingriff, Operationsrisiko, Operationsdauer > 2 h, erste präoperative bzw. nach Anästhesieausleitung letzte im OP gemessene S_p_O_2_ < 97 %, intraoperative Beatmungsparameter (inspiratorischer Spitzendruck > 30 mbar, F_I_O_2_ > 50 %, „driving pressure“ > 15 mbar, PEEP > 5 mbar), Gabe eines Relaxans oder eines lang wirksamen Opioids sowie Durchführung einer totalen intravenösen Anästhesie (TIVA). Ein nichtsignifikanter Hosmer-Lemeshow-Test wurde für die Güte des Regressionsmodells vorausgesetzt und Odds ratio und 95 %-Konfidenzintervall berechnet.

Das PSM wurde mittels MatchIt® package für R [12] durchgeführt und durch „standardized mean deviation“ (SMD) überprüft, wobei SMD < 0,1 als ausgeglichene Balance gilt.

Die statistische Auswertung von Unterschieden zwischen den Gruppen (OM vs. MM) erfolgte mittels *t*-Test für gepaarte Stichproben, Wilcoxon-Rangsummentest oder McNemar-Chi-Quadrat-Test.

## Ergebnisse

**Figure 1:**
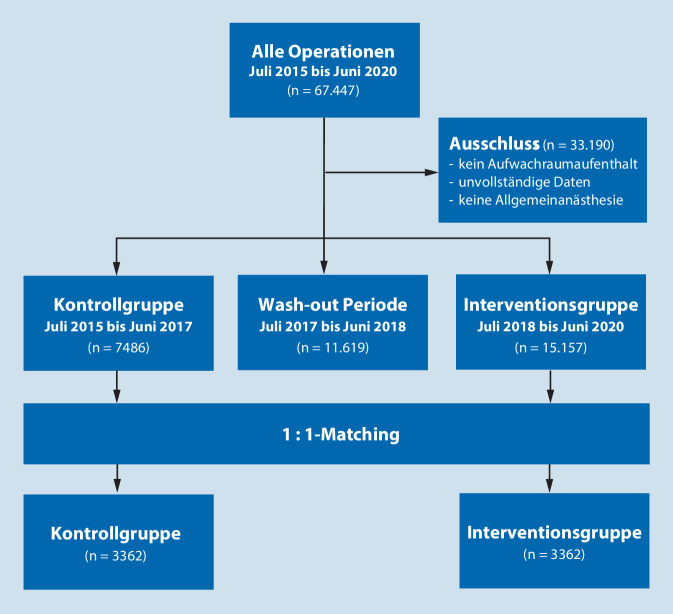

Insgesamt wurden 67.447 Datensätze extrahiert. Davon konnten nach Anwendung der Ein- und Ausschlusskriterien sowie Entfernung der Wash-out-Periode 22.638 Datensätze (ungematchte Gesamtkohorte) eingeschlossen werden. Die Gruppengrößen nach PSM betrug jeweils 3362 Datensätze/Gruppe (Abb. 1).

**Figure 2:**
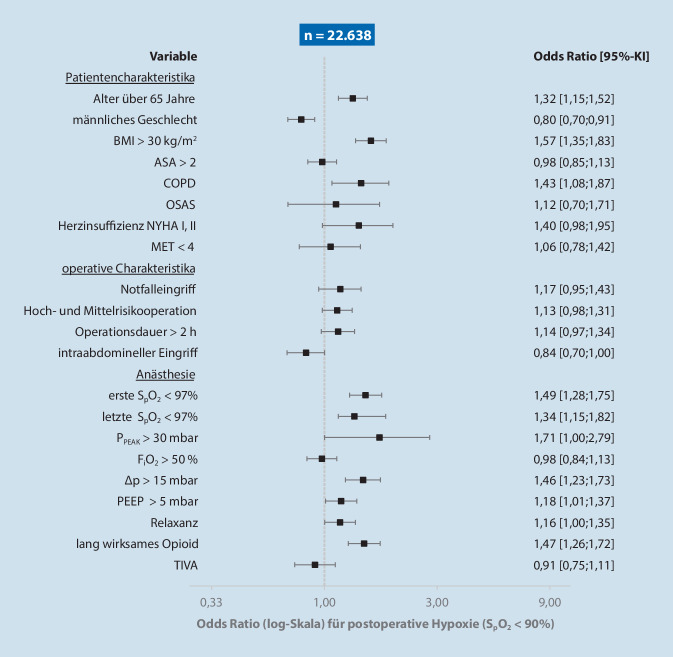

Die demografischen und prozedurassoziierten Daten der ungematchten Kohorte als auch gematchten Gruppen sind in Tab. 1 dargestellt. Die Risikofaktoren für das Auftreten einer Hypoxämie (S_p_O_2_ < 90 %) in der ungematchten Gesamtkohorte zeigt Abb. 2 mit einer erniedrigten präoperativen S_p_O_2_ (OR 1,49 [1,28; 1,75]) als stärkstem Einflussfaktor. Mindestens ein Risikofaktor für Hypoxämie lag bei 90 % der Patienten vor.

**Table 1:** 

	Ungematched			Gematched		
	OM (*n* = 7481)	MM (*n* = 15.157)	SMD	OM (*n* = 3362)	MM (*n* = 3362)	SMD
**Alter, Jahre**	55 [35;75]	58 [38;78]	0,141	58 [40;76]	58 [40;76]	< 0,001
**Männliches Geschlecht**	3723 (50 %)	8499 (56 %)	0,127	1754 (52 %)	1754 (52 %)	< 0,001
**Gewicht, kg**	75 (20)	77 (10)	0,087	76 (18)	77 (18)	0,006
**Größe, cm**	170 (13)	172 (18)	0,042	171 (10)	173 (45)	0,046
**BMI, kg/m**^**2**^			0,056			< 0,001
Unter 25	3580 (48 %)	6828 (45 %)		1574 (47 %)	1574 (47 %)	
25–30	2464 (33 %)	5238 (35 %)		1165 (35 %)	1165 (35 %)	
Über 30	1437 (19 %)	3091 (20 %)		623 (19 %)	623 (19 %)	
**KHK**	528 (7 %)	1096 (7 %)	0,007	265 (8 %)	211 (6 %)	0,064
**NYHA I und II**	192 (3 %)	319 (2 %)	0,031	83 (3 %)	53 (2 %)	0,066
**CNI**	107 (1 %)	270 (2 %)	0,028	47 (1 %)	34 (1 %)	0,037
**COPD**	242 (3 %)	521 (3 %)	0,011	122 (4 %)	104 (3 %)	0,061
**IDDM**	209 (3 %)	472 (3 %)	0,019	109 (3 %)	90 (3 %)	0,036
**TIA oder Schlaganfall**	263 (4 %)	541 (4 %)	0,003	126 (4 %)	111 (3 %)	0,026
**ASA-Klassifikation**			0,196			< 0,001
1	2022 (27 %)	3399 (22 %)		742 (22 %)	742 (22 %)	
2	4118 (55 %)	7886 (52 %)		2045 (61 %)	2045 (60 %)	
3	1310 (18 %)	3748 (25 %)		574 (17 %)	574 (17 %)	
4	30 (0 %)	124 (1 %)		1 (0 %)	1 (0 %)	
**N‑Klassifikation**			0,103			< 0,001
N1	39 (0 %)	43 (0 %)				
N2	144 (2 %)	225 (2 %)		3 (0 %)	3 (0 %)	
N3	182 (2 %)	427 (3 %)		16 (1 %)	16 (1 %)	
N4	232 (3 %)	720 (5 %)		32 (1 %)	32 (1 %)	
N5	6879 (92 %)	13742 (91 %)		3311 (99 %)	3311 (99 %)	
**Abdomineller Eingriff**	1378 (18 %)	2319 (15 %)	0,083	625 (19 %)	424 (13 %)	0,165
**Eingriff im Kopf-Hals-Bereich**	2763 (37 %)	5302 (35 %)	0,041	1261 (38 %)	1294 (39 %)	0,020
**Intrakranieller Eingriff**	218 (3 %)	577 (4 %)	0,050	98 (3 %)	178 (5 %)	0,120
**Intrathorakaler Eingriff**	38 (1 %)	58 (0 %)	0,019	17 (1 %)	19 (1 %)	0,008
**Operationsrisiko**			0,062			< 0,001
Hoch	88 (1 %)	294 (2 %)		15 (0 %)	15 (0 %)	
Mittel	3881 (52 %)	7749 (50 %)		1805 (54 %)	1805 (54 %)	
Niedrig	3512 (47 %)	7114 (47 %)		1542 (46 %)	1542 (46 %)	
**Fachdisziplin**			0,456			0,410
Allgemeinchirurgie	785 (11 %)	1753 (12 %)		309 (9 %)	298 (9 %)	
Gefäßchirurgie	938 (13 %)	1943 (13 %)		81 (2 %)	135 (4 %)	
Gynäkologie	682 (9 %)	59 (0 %)		278 (8 %)	34 (1 %)	
HNO	1074 (14 %)	2290 (15 %)		434 (13 %)	559 (17 %)	
Kieferchirurgie	619 (8 %)	1005 (7 %)		256 (8 %)	217 (7 %)	
Neurochirurgie	674 (9 %)	1763 (12 %)		303 (9 %)	488 (15 %)	
Ophthalmochirurgie	938 (13 %)	1943 (13 %)		476 (14 %)	463 (14 %)	
Unfallchirurgie	1404 (19 %)	2722 (18 %)		687 (20 %)	636 (19 %)	
Urologie	1057 (14 %)	2735 (18 %)		535 (16 %)	531 (16 %)	

*OM* ohne Transport-Monitoring, *MM* mit Transport-Monitoring, *BMI* Body-Mass-Index, *KHK* koronare Herzkrankheit, *CNI* chronische Niereninsuffizienz, *COPD* chronisch obstruktive Lungenerkrankung, *IDDM* insulinabhängiger Diabetes mellitus, *TIA* transitorische ischämische Attacke, *ASA* körperlicher Status gemäß Einteilung der American Society of Anesthesiologists, *N‑Klassifikation* Klassifikation von Notfalloperationen nach BDA/DGAI, *Operationsrisiko* operationsassoziiertes kardiales Risiko nach DGAI

Nach anschließender PSM war die Verteilung der gematchten Variablen sowie der Komorbiditäten zwischen MM- und OM-Datensätzen ausgeglichen (SMD < 0,1). Imbalancen (SMD > 0,1) lagen bei Eingriffslokalisation und den operativen Disziplinen vor (Tab. 1) sowie bei Narkoseform (TIVA/balancierte Anästhesie), Relaxanziengabe, Driving pressure und mittlerer arterieller Druck (MAD) (Tab. 2). Die erste präoperativ gemessene S_p_O_2_ unterschied sich zwischen den Gruppen nicht (MM, OM: 98 % [96; 100], SMD = 0,005). Während des Transports erhielten beide Gruppen gleich häufig Sauerstoff via Nasenbrille oder Maske (MM: 933 (3 %), OM: 943 (3 %), SMD = 0,051). Der Gesamttransportprozess war in der Gruppe ohne Monitor länger als in der Gruppe mit Monitor (OM: 10 min [8; 12], MM: 8 min [5; 10], SMD = 0,536) trotz unveränderter Infrastruktur des ZOP.

**Table 2:** 

	OM (*n* = 3362)	MM (*n* = 3362)	SMD
**Narkoseform**			
TIVA	1438 (43 %)	890 (27 %)	0,348
Balancierte Anästhesie	1924 (57 %)	2472 (63 %)	
**Medikamente**			
Fentanyl, µg	400 [300; 500]	400 [300; 500]	0,109
Remifentanil, µg	1120 [746; 1780]	1151 [763; 1960]	0,082
Sufentanil, µg	50 [48; 53]	40 [30; 60]	0,057
Propofol, mg/kg	5 [3; 10]	4 [3; 9]	0,048
Rocuronium, mg	50 [40; 80]	70 [50; 80]	0,504
Mivacurium, mg	16 [13; 20]	15 [12; 18]	0,196
Cisatracurium, mg	10 [9; 15]	10 [8; 14]	0,134
**Regionalanästhesie**			
Alleinige Allgemeinanästhesie	3302 (98 %)	3309 (98 %)	0,018
Allgemeinanästhesie + Epiduralanästhesie	37 (1 %)	34 (1 %)	
Allgemeinanästhesie + Regionalanästhesie	23 (< 1 %)	19 (< 1 %)	
**Beatmung**			
PEEP, mbar	5 (±2)	5 (±2)	0,021
p_peak_, mbar	16 [14; 19]	16 [14; 18]	0,104
∆p, mbar	11 [9; 14]	11 [9; 13]	0,116
F_I_O_2_, %	43 [38; 51]	44 [37; 52]	0,006
**Vitalparameter präoperativ**			
Erste S_p_O_2_, %	98 [96; 100]	98 [96; 100]	0,005
Erster MAD, mm Hg	90 (±13)	96 (±13)	0,468
Erste HF, min^−1^	74 [65; 83]	73 [65; 83]	0,030
**Vitalparameter intraoperativ**			
Letzter MAD, mm Hg	86 (±17)	90 (±18)	0,271
Letzte HF, min^−1^	70 [59; 85]	71 [59; 85]	0,008
Mittlere intraoperative Körpertemperatur, °C	36 (±1)	36 (±1)	0,097
**Vitalparameter Aufwachraum**			
Erster MAD, mm Hg	85 (±15)	92 (±16)	0,395
Erste HF, min^−1^	80 [64; 96]	80 [64; 96]	0,023
**Gesamttransportprozess, min**	10 [8; 12]	8 [5; 10]	0,536
**Transport in den AWR ohne Sauerstoff,** ***n*** **(%)**	2429 (97 %)	2419 (97 %)	0,051
**Anästhesiedauer, h**	2 [1; 3]	2 [1; 3]	0,033
**Operationsdauer, h**	1 [1; 2]	1 [1; 2]	0,029

*OM* ohne Transport-Monitoring, *MM* mit Transport-Monitoring,* TIVA* totale intravenöse Anästhesie, *PEEP* „positive endexpiratory pressure“, *p*_*peak*_ Beatmungsspitzendruck, *∆p* „driving pressure“ = p_peak_ – PEEP, *F*_*I*_*O*_*2*_ inspiratorische Sauerstoffkonzentration, *MAD* mittlerer arterieller Druck, *HF* Herzfrequenz

**Figure 3:**
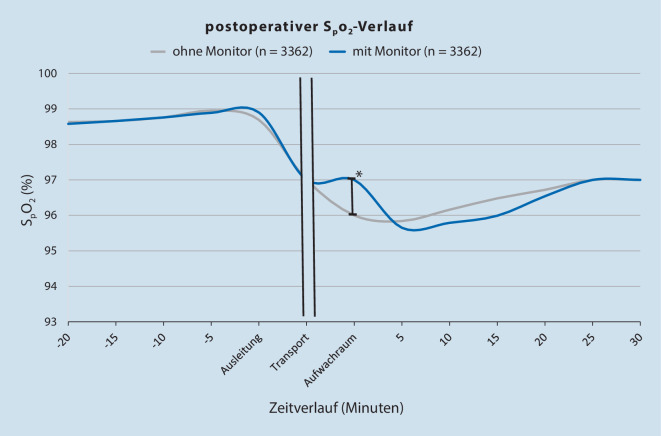

Nach PSM (OM vs. MM) war die initiale S_p_O_2_ im AWR mit TM höher (MM: 97 % [94; 99], OM: 96 % [94; 99], *p* < 0,001), zeigte dann ab 5 min Aufenthalt im AWR und im weiteren Verlauf keine Unterschiede mehr (Abb. 3). Die Differenz von initialer S_p_O_2_ im AWR zum präoperativ gemessenen S_p_O_2_-Wert ebenfalls mit TM niedriger (MM: –1 % [−3; 1], OM: –2 % [−4; 0], *p* < 0,001). Die separate Analyse der Patienten ohne einen Risikofaktor (*n* = 680) zeigte keinen Unterschied in der S_p_O_2_ mit und ohne TM (MM: 99 [97; 100] %, OM: 98 [97; 100] %, *p* = 0,393), wobei bei Patienten mit mehr als einem Risikofaktor (*n* = 6044) der initiale S_p_O_2_-Wert mit TM höher war (MM: 97 [94; 99] %, OM: 96 [94; 98] %, *p* < 0,001, Tab. 3). Hypoxämien traten in beiden Gruppen gleich häufig (MM, OM: 5 %, *p* = 0,755) auf, was sich auch bei steigender Anzahl von Risikofaktoren nicht änderte: ohne Risikofaktoren 0 % (*p* = 1,000), mit mehr als einem Risikofaktor 5 % (*p* = 0,815, Tab. 3). Der Aldrete-Score bei AWR-Aufnahme und die Atemfrequenz zeigten ebenfalls Unterschiede, wohingegen die Krankenhausverweildauer als auch die Häufigkeit der anschließenden Aufnahme auf die Intensivstation unbeeinflusst waren (Tab. 3).

**Table 3:** 

	OM (*n* = 3362)	MM (*n* = 3362)	*p*-Wert
**Initiale S**_**p**_**O**_**2**_** im AWR, %**			
Gesamt (*n* = 6724)	96 [94; 99]	97 [94; 99]	< 0,001
0 Risikofaktoren ^a^ (*n* = 680)	99 [97; 100]	98 [97; 100]	0,393
≥1 Risikofaktor ^a^ (*n* = 6044)	96 [94; 98]	97 [94; 99]	< 0,001
*∆S*_*p*_*O*_*2*_, %	−2 [0; −4]	−1 [1; −3]	< 0,001
**Hypoxämie, *n* (%)**			
Gesamt	150 (5 %)	161 (5 %)	0,755
0 Risikofaktoren ^a^	3 (0 %)	2 (0 %)	1,000
≥ 1 Risikofaktor ^a^	148 (5 %)	159 (5 %)	0,815
**Schweregrad Hypoxämie, *n* (%)**			
Mild (S_p_O_2_ 90–86 %)	110 (3 %)	118 (3 %)	0,985
Moderat (S_p_O_2_ 86–81 %)	29 (1 %)	32 (1 %)	
Schwer (S_p_O_2_ < 81 %)	13 (< 1 %)	13 (< 1 %)	
**Erste Atemfrequenz, min**^**−1**^	14 [11; 17]	15 [12; 18]	< 0,001
**Aldrete-Score > 8, *n* (%)**	2665 (81 %)	2830 (83 %)	0,004
**Krankenhausverweildauer, Tage**	4 [2; 7]	4 [2; 7]	0,109
**Postoperative ITS-Aufnahme, *n* (%)**	48 (1 %)	41 (1 %)	0,400

*OM* ohne Transport-Monitoring, *MM* mit Transport-Monitoring,* ∆S*_*p*_*O*_*2*_ Differenz der initialen Sättigung im AWR zur ersten perioperativen Messung

^a^ *Risikofaktoren:* Alter > 65 Jahre, BMI > 30 kg/m^2^, COPD, erster präoperativer S_p_O_2_ < 97 %, letzter intraoperativer S_p_O_2_ < 97 %, ∆p > 15 mbar, PEEP > 5 mbar, lang wirksames Opioid

## Diskussion

In dieser retrospektiven Datenbankanalyse konnten 8 Risikofaktoren für Hypoxämien nach einer Allgemeinanästhesie identifiziert werden, wovon eine präoperative reduzierte S_p_O_2_ < 97 % den größten Einfluss hatte. Außerdem gab es Hinweise darauf, dass ein Transport-Monitoring (TM) mit einer höheren initialen S_p_O_2_ sowie höherem Aldrete-Score im AWR assoziiert war. Dabei war der Anteil von Patienten mit kritischer Hypoxämie in beiden Gruppen gleich.

### Risikofaktoren für Hypoxämie

In den letzten 10 Jahren wurden nur wenige Publikationen mit sehr uneinheitlichen Ergebnissen zu Risikofaktoren für eine initiale postoperative Hypoxämie im AWR veröffentlicht [4, 14, 22, 23]. Diese früheren Ergebnisse zeigten, dass die ersten Minuten nach der Extubation eine kritische Phase für Hypoxämien darstellen [22], was auch durch diese Arbeit erneut bestätigt wird. Dabei auftretende modifizierbare Risikofaktoren waren eine intraoperative Gabe lang wirksamer Opioide, eine hohe Dosis an Neostigmin, hohe intraoperative F_I_O_2_ sowie niedrige F_I_O_2_ kurz vor der Extubation [22]. Eine weitere Arbeit an 970 Patienten verdeutlichte das Auftreten von Hypoxämien in 17 % der Fälle und diesbezüglich eine Assoziation mit hohem Alter, BMI, präoperativer S_p_O_2_ < 97 %, Wahl des Opioids, Driving pressure (∆p), volumenkontrollierter Beatmung und Lachgas [4]. Auch Labaste et al. wiesen an 505 prospektiv untersuchten Patienten in 13 % der Fälle Hypoxämien nach, welche zusätzlich mit einem hohem Sedierungsgrad sowie einer letzten S_p_O_2_ < 97 % im OP assoziiert waren [14]. Eine weitere prospektive Observationsstudie von 2013 ermittelte Sedierungsgrad, Atemfrequenz und Transport ohne Sauerstoff als weitere Risikofaktoren für Hypoxämien nach Allgemeinanästhesie mit einer Häufigkeit von 19 % in der Gesamtkohorte und nur 0,8 % unter Sauerstoffapplikation [23].

Im Vergleich zu diesen älteren Arbeiten fanden spezifische Narkoseformen wie die Supplementierung von Lachgas im hier ausgewerteten Patientenkollektiv nicht statt. Auch können moderne Anpassungen bei Anästhesieverfahren wie z. B. die Einführung kurz wirksamer Hypnotika, Analgetika und Relaxanzien sowie verbesserte Operationstechniken und die Abkehr von einer präoperativen Benzodiazepingabe einen Einfluss haben [19]. Daher sind diese Erkenntnisse nicht allumfassend auf heutige Verfahren übertragbar. Die Ergebnisse der vorliegenden Untersuchung zeigen neben patientenspezifischen Faktoren (Alter > 65 Jahre, BMI > 30 kg/m^2^, chronisch obstruktive Lungenerkrankung (COPD)) die Relevanz von anästhesiespezifischen Daten (∆p > 15 mbar, PEEP > 5 mbar, intraoperative Gabe eines lang wirksamen Opioids) sowie von perioperativen Vitalwerten (erste präoperative S_p_O_2_ < 97 %, nach Anästhesieausleitung letzte im OP gemessene S_p_O_2_ < 97 %) für die Abschätzung des postoperativen Hypoxämierisikos.

### Patientenspezifische Risikofaktoren

Hohes Alter wird in der Literatur durchweg als Risikofaktor im Zusammenhang mit postoperativen S_p_O_2_-Abfällen und postoperativen pulmonalen Komplikationen (PPC) genannt [4, 16, 24], was unsere Ergebnisse erneut bestätigen. Ebenso bestätigen unsere Ergebnisse eine Adipositas (BMI > 30 kg/m^2^) als Risikofaktor für Hypoxämien [4, 14], was möglicherweise durch ein vermindertes pulmonales Residualvolumen oder auch Begleiterkrankungen wie ein obstruktives Schlafapnoesyndrom erklärbar ist.

### Anästhesiespezifische Risikofaktoren

∆p > 15 mbar ist ein bekannter Risikofaktor für PPC [15], und sowohl Aust et al. als auch unsere Ergebnisse bestätigen dies als unabhängigen Risikofaktor für eine postoperative Hypoxämie [4]. Ebenso scheint die Höhe des intraoperativen PEEP mit der Häufigkeit von Hypoxämien assoziiert zu sein. Zwar zeigen Arbeiten mit höherem PEEP im Sinne von Rekrutierungsmanövern einen positiven Effekt auf die Oxygenierung [7]. Im Gegensatz dazu könnte aber die Notwendigkeit eines kontinuierlich erhöhten PEEP (> 5 mbar) auch Ausdruck einer bestehenden pulmonalen Störung sein und konnte in der vorliegenden Arbeit als Risikofaktor identifiziert werden. Neben der Respiratoreinstellung ließen sich mittel- bis lang wirksame Opioide als spezifischer Risikofaktor des anästhesiologischen Managements identifizieren, welches auch in früheren Arbeiten bestätigt wurde [5, 22]. Hierbei ist erwähnenswert, dass dies durch die Anesthesia Patient Safety Foundation mit einer Forderung von obligater Pulsoxymetrie nach Narkose mit Opioiden adressiert ist [27].

### Perioperative Messwerte als Risikofaktoren

Die verminderte präoperative S_p_O_2_ (< 97 %) zeigt in dieser Arbeit die stärkste Assoziation als Risikofaktor für eine postoperative Hypoxämie. Die Assoziation von niedriger präoperativer S_p_O_2_ mit einer Hypoxämie und PPC ist zwar ebenfalls in älteren Arbeiten adressiert [4, 8]. Dass dies in den hier vorliegenden Daten der Risikofaktor mit der stärksten Assoziation ist, betont jedoch erneut die Relevanz dieses einfach zu erhebenden und damit ubiquitär nutzbaren Messwertes. Neben der initialen S_p_O_2_ zeigte auch eine nach Anästhesieausleitung letzte im OP gemessene S_p_O_2_ < 97 % eine vorhandene Risikokorrelation für das Auftreten von Hypoxämien an. Demnach könnten neben den schon oben erwähnten Forderungen eines Monitorings nach Opioidnutzung dies auch bei initialer und/oder nach Ausleitung reduzierter S_p_O_2_ sinnvoll sein. Ein Transport ohne Sauerstoffapplikation scheint ebenfalls ein Risikofaktor zu sein [23]. Da jedoch eine undifferenzierte Sauerstoffapplikation ebenso negative Effekten haben kann, sollte diese nicht unkritisch angewendet werden [9, 17, 25]. In der hier vorliegenden Arbeit wurde in 97 % ohne Sauerstoffapplikation transportiert, und trotzdem traten im Vergleich zu vorherigen Arbeiten deutlich weniger Hypoxämien auf [4, 14].

### Eingriffsspezifische Risikofaktoren

Operations‑/Anästhesiedauer, Operationsrisiko, Dringlichkeit sowie Lokalisation (intraabdominell vs. nichtintraabdominell) hatten in unserer Analyse keinen Einfluss auf das Auftreten einer postoperativen Hypoxämie. In einer Observationsstudie von Xue et al. 1999 war eine thorakoabdominelle oder abdominelle Eingriffslokalisation mit postoperativer Hypoxämie assoziiert [28]. Dass dies in unseren Ergebnissen und den oben genannten Studien der letzten Dekade nicht der Fall ist, könnte u. a. an den in den letzten Jahrzehnten verbesserten Operationstechniken oder der besseren Identifizierung von Risikopatienten mit direkter Verlegung aus dem OP in eine intensivmedizinische Einheit liegen.

### Einfluss des Transport-Monitorings

Aktuelle Studien, die den Einfluss des Transport-Monitorings auf die S_p_O_2_ im AWR untersuchen, fehlen bislang. Die größte Studie, die sich mit der perioperativen Verwendung der Pulsoxymetrie beschäftigte, war von Moller et al. 1993 an 20.802 Patienten. Hier wurde eine Hypoxämie intraoperativ und im AWR mittels Pulsoxymetrie 19-mal häufiger detektiert als ohne Pulsoxymetrie [18]. In einer Umfrage von Aust et al. im Jahr 2012 unter deutschen Universitätskliniken gaben 80 % an, kein postoperatives TM einzusetzen [4]. Auch die aktuell gültige Empfehlung schreibt dies nicht vor [3]. Unsere Ergebnisse zeigen, dass die S_p_O_2_ bei Ankunft im AWR – bei gleicher präoperativer S_p_O_2_ – mit TM höher war als ohne. Ebenso zeigten Patienten mit TM einen höheren Aldrete-Score bei Ankunft im AWR. Ob die detektierten Unterschiede zwischen beiden Gruppen zu einer Beeinflussung des weiteren Behandlungsverlaufes geführt haben, kann mit den hier vorliegenden Daten nicht abschließend beantwortet werden. Eine Assoziation des TM mit reduzierter Krankenhausverweildauer, Anzahl an Hypoxämie oder ungeplanten ITS-Aufnahmen war in unseren Ergebnissen nicht zu sehen. Da aber TM mit höherer S_p_O_2_ und höherem Aldrete-Score assoziiert scheint, wäre es naheliegend, die Anwendung für den Transport zwischen OP und AWR zu empfehlen.

### Limitationen

Die Limitationen der Studie ergeben sich u. a. aus dem retrospektiven Charakter und der Datenerhebung aus klinischen Routinedaten. Die Ergebnisse sind als rein deskriptiv zu verstehen, da keine Power-Analyse durchgeführt und nicht für multiples Testen adjustiert wurde. Da die Behandlungen der Gruppen nach PSM zu verschiedenen Zeitpunkten stattfanden, liegen trotz Matching Imbalancen bestimmter anästhesie- und eingriffsspezifischer Daten vor. Aufgrund von klinikinternen Änderungen wurden in der MM-Gruppe mehr balancierte Anästhesien statt TIVA durchgeführt, die Menge an gegebenem Rocuronium war höher, und es fanden weniger gynäkologische Eingriffe statt. Da jedoch weder die Anästhesieform (balancierte Anästhesie vs. TIVA) noch die Relaxansgabe oder die Eingriffslokalisation in der Regressionsanalyse einen Einfluss auf das Auftreten von Hypoxämie hatten, ist der Einfluss auf den Gruppenvergleich OM vs. MM vermutlich vernachlässigbar. Die unterschiedliche Dauer des gesamten Transportprozesses mit oder ohne Monitoring ist eine weitere zu diskutierende Limitation. Hier kann aufgrund des retrospektiven Studiendesigns nicht abschließend geklärt werden, ob alleinig das Ablegen und erneute Anbringen des Monitorings zur längeren Prozesszeit der OM Gruppe geführt hat, oder ob möglicherweise auch (diskrete) Prozessänderungen im täglichen OP-Ablauf zu verkürzter Transportzeit und besserer Oxygenierung der MM-Gruppe beigetragen haben könnten.

## Fazit für die Praxis

- Die vorliegende Studie konnte 8 Risikofaktoren für eine initiale Hypoxämie im AWR bestimmen: Alter > 65 Jahre, BMI > 30 kg/m^2^, COPD, ∆p > 15 mbar, PEEP > 5 mbar, intraoperative Gabe eines lang wirksamen Opioids, erste präoperative S_p_O_2_ < 97 %, nach Anästhesieausleitung letzte im OP gemessene S_p_O_2_ < 97 %.
- 90 % aller Patienten hatten mindestens einen Risikofaktor für eine postoperative Hypoxämie.
- Bei Vorliegen von Risikofaktoren geht die Verwendung von Transport-Monitoring mit einer geringeren Desaturierung auf dem Weg in den AWR einher.
- Insbesondere eine präoperative S_p_O_2_ < 97 % ist mit postoperativer Hypoxämie assoziiert und sollte daher zu vermehrter Vigilanz führen.

## Supplementary Information




