# Supplementary material for: Risk factors for postoperative hypoxemia during transport to the postanesthesia care unit and influence of transport monitoring: A retrospective propensity score-matched databank analysis
Source: Anaesthesiologie. 2023 Jun 9;72(7):488–97. [Article in German] doi: 10.1007/s00101-023-01296-y (PMC10322755; doi:10.1007/s00101-023-01296-y)
Supplement: Supplementary file 1 [file 101_2023_1296_MOESM1_ESM.pdf]

Zusatzmaterial zum Beitrag **„Risikofaktoren für postoperative Hypoxämie während des Transportes in den Aufwachraum und Einfluss von Transportmonitoring. Eine retrospektive propensity-score gematchte Datenbankanalyse"** von Haller K, Trauzeddel RF, Treskatsch S et al. (2023) in *Die Anaesthesiologie.*
Beitrag und Zusatzmaterial stehen Ihnen auf www.springermedizin.de zur Verfügung. Bitte geben Sie dort den Beitragstitel in die Suche ein.

---

Anhang Tabelle 1: Binäre logistische Regressionsanalyse für initiale Hypoxämie (SpO2 ≤ 90%) im Aufwachraum an 22.638 Patienten.

| | Regressionskoeffizient | Standardfehler | p-Wert | Odds Ratio | 95 % Konfidenzintervall | |
|---|---|---|---|---|---|---|
| | | | | | oberer Wert | unterer Wert |
| (Intercept) | -3.943 | 0.134 | < 0,001 | | | |
| Alter > 65 | 0.339 | 0.069 | < 0,001 | 1,32 | 1,15 | 1,52 |
| Männliches Geschlecht | -0.232 | 0.067 | < 0,001 | 0,80 | 0,70 | 0,91 |
| BMI > 30 kg/m² | 0.476 | 0.076 | < 0,001 | 1,57 | 1,35 | 1,83 |
| ASA > 2 | -0.021 | 0.071 | 0.766 | 0,98 | 0,85 | 1,13 |
| COPD | 0.378 | 0.142 | 0.008 | 1,43 | 1,08 | 1,87 |
| OSAS | 0.131 | 0.227 | 0.566 | 1,12 | 0,70 | 1,71 |
| NYHA I und II | 0.343 | 0.176 | 0.050 | 0,98 | 1,40 | 1,95 |
| < 4 MET | 0.070 | 0.153 | 0.648 | 1,06 | 0,78 | 1,42 |
| Notfalleingriff | 0.163 | 0.105 | 0.121 | 1,17 | 0,95 | 1,43 |
| OP-Risiko hoch, mittel | 0.137 | 0.074 | 0.065 | 1,13 | 0,98 | 1,31 |
| OP-Dauer > 2 Stunden | 0.137 | 0.082 | 0.096 | 1,14 | 0,97 | 1,34 |
| intraabdomineller Eingriff | -0.166 | 0.093 | 0.075 | 0,84 | 0,70 | 1,00 |
| erste SpO2 < 97 % | 0.413 | 0.080 | < 0,001 | 1,49 | 1,28 | 1,75 |
| letzte SpO2 < 97 % | 0.296 | 0.079 | < 0,001 | 1,34 | 1,15 | 1,82 |
| $P_{PEAK}$ > 30 mbar | 0.536 | 0.260 | 0.039 | 1,71 | 1,00 | 2,79 |
| FiO2 > 50 % | -0.021 | 0.074 | 0.776 | 0,98 | 0,84 | 1,13 |
| Driving Pressure > 15 mbar | 0.385 | 0.087 | < 0,001 | 1,46 | 1,23 | 1,73 |
| PEEP > 5 mbar | 0.166 | 0.078 | 0.033 | 1,18 | 1,01 | 1,37 |
| Relaxanz | 0.150 | 0.076 | 0.049 | 1,16 | 1,00 | 1,35 |
| langwirksames Opioid | 0.382 | 0.079 | < 0,001 | 1,47 | 1,26 | 1,72 |
| TIVA | -0.094 | 0.101 | 0.355 | 0,91 | 0,75 | 1,11 |
| Hosmer-Lemeshow-Test: $\chi^2$ (4) = 8, p = 0,8 | | | | | | |
